# Metallic θ-phase tantalum nitride has a thermal conductivity triple that of copper

**DOI:** 10.1126/science.aeb1142

**Published:** 2026-01-15

**Authors:** Suixuan Li, Chuanjin Su, Zihao Qin, Ahmet Alatas, Martin Kunz, Takahiro Yamada, Shelly D. Kelly, Mary H. Upton, Anthony Gironda, Jiyong Zhao, Bora Kalkan, Wanli Yang, Toshihiro Aoki, Yongjie Hu

**Affiliations:** 1 Department of Mechanical and Aerospace Engineering, University of California, Los Angeles, Los Angeles, CA, USA.; 2 Advanced Photon Source, Argonne National Laboratory, Argonne, IL, USA.; 3 Advanced Light Source, Lawrence Berkeley National Laboratory, Berkeley, CA, USA.; 4 Institute of Multidisciplinary Research for Advanced Material, Tohoku University, 2-1-1 Katahira, Aoba-ku, Sendai, Japan.; 5 Irvine Materials Research Institute, Irvine, CA, USA.; 6 California NanoSystems Institute, Los Angeles, CA, USA.; 7 Department of Materials Science and Engineering, University of California, Los Angeles, Los Angeles, CA, USA.; 8 Center for Quantum Science and Engineering, University of California, Los Angeles, Los Angeles, CA, USA.

## Abstract

Efficient heat dissipation is fundamentally limited by intrinsic scattering mechanisms that cap the thermal conductivity of metallic materials such as copper to ~400 watts per meter-kelvin. Here we report the experimental realization of single-crystalline θ-phase tantalum nitride (θ-TaN), a metastable transition metal nitride predicted to overcome this limitation. We measured a room-temperature thermal conductivity of ~1100 watts per meter-kelvin, nearly three times that of copper. Synchrotron-based inelastic x-ray scattering revealed a distinctive phonon band structure with a large acoustic-optical gap and phonon bunching, which suppress phonon-phonon scattering. Ultrafast optical spectroscopy confirmed exceptionally weak electron-phonon coupling and validated first-principles calculations. These findings redefine the thermal transport limits of metallic materials and open new opportunities for advancing thermal management in electronics and power systems.

## Editor’s summary

Single crystals of the #-phase of tantalum nitride have an ultrahigh thermal conductivity, consistent with previous theoretical predictions. Li *et al*. used time-domain thermoreflectance to show that their samples had a thermal conductivity about three times greater than that of copper. Inelastic x-ray scattering revealed that phonon-phonon scattering, which reduces thermal conductivity, was suppressed. —Phil Szuromi

Efficient thermal management is essential for sustaining performance, reliability, and energy efficiency in many types of electronic devices, driving the search for materials with high thermal conductivity capable of rapidly extracting heat from localized hotspots (1–5). The high thermal conductivity of metallic materials has enabled their widespread use as heat sinks, spreaders, interconnects, packaging substrates, and structural components in heat pipes and vapor chambers. For example, copper, with a thermal conductivity of ~400 W/m·K, accounts for ~30% of the global heat sink materials market and remains the dominant choice for high-performance cooling substrates used with computer chips and artificial intelligence accelerators (6). However, a thermal conductivity near 400 W/m·K has remained the upper limit for metallic heat transport for more than a century.

This thermal conductivity limit arises from fundamental physics governing the interplay of electron and phonon transport. Inherently, in typical metals and semimetals, thermal conductivity is constrained by electron-phonon interactions and phonon anharmonicity associated with a soft metallic lattice (7–9). Electron-phonon coupling induces frequent scattering events in which conduction electrons exchange energy and momentum with lattice vibrations, reducing both the electronic and lattice contributions to heat transport. Concurrently, anharmonic phonon-phonon interactions decrease phonon lifetimes, further diminishing the lattice contribution. Together, these intrinsic scattering mechanisms have historically precluded metallic materials from reaching ultrahigh thermal conductivity.

Recent advances in first-principles theory have shown excellent agreement with the measured thermal conductivity of a wide range of materials (10–24), including silicon, diamond, boron nitride (BN), boron arsenide (BAs), and boron phosphide (BP). Such theoretical works provide new physical insights into the nature of thermal transport and motivate the exploration of high thermal conductivity mechanisms in new materials, including potential unexplored metallic analogs. Indeed, some very recent first-principles studies challenge the long-standing metallic limit and point to a possible breakthrough (25–27): Theoretical predictions indicate that a metastable transition metal nitride, the θ-phase of tantalum nitride (θ-TaN), may exhibit record-high thermal conductivity among all metallic materials, potentially surpassing that of copper and silver and approaching that of diamond or BAs (14). This extraordinary behavior is attributed to an unusual combination of ultrastiff atomic bonding, weak phonon anharmonicity, and anomalously low electron-phonon coupling, suggesting that θ-TaN may inaugurate a new class of metallic materials with ultrahigh thermal conductivity previously thought inaccessible.

Experimental efforts to synthesize and characterize θ-TaN have been very scarce so far. Although the growth of θ-TaN through high-pressure solid-phase conversion was reported as early as the 1950s, detailed structural characterizations and property measurements were not provided (28–31). Thermodynamic analysis of the Ta-N phase diagram suggests that the stabilization of the θ-phase under high-pressure and high-temperature conditions may be driven by displacement of Ta atoms from trigonal planar coordination sites (32–34). However, the intrinsic difficulty of stabilizing transition metal nitrides under such extreme conditions, which requires several gigapascals of pressure and temperatures of several thousand kelvin, has made crystal synthesis of θ-TaN particularly challenging (35–37). The TaN system is further complicated by the existence of multiple competing polymorphs, including cubic (δ-TaN), hexagonal (ε-TaN), and tetragonal (t-TaN) phases (38, 39). As a result, most reported efforts have yielded samples containing grain boundaries, defects, and mixed phases, and these structural imperfections degrade transport properties (40, 41) to levels far below theoretical expectations (25–27). Subsequent theoretical analyses indicate that defect scatterings play a dominant role, making it impossible to probe the intrinsic thermal transport in the absence of high-quality θ-TaN crystals. Defect-induced electron-phonon scattering, along with enhanced phonon scattering at grain boundaries and phase interfaces, can substantially reduce the thermal conductivity.

## Single-crystalline θ-TaN synthesis and structural characterization

High quality θ-TaN single crystals were synthesized through a flux-assisted metathesis reaction (see supplementary materials) in which sodium served both as a reducing agent and a flux to facilitate the nitridation of tantalum oxides in a nitrogen-rich environment. This flux-mediated approach overcame the synthesis challenges of conventional high-pressure, high-temperature routes and resulted in improved crystallinity, enhanced phase purity, well-faceted grain morphology, and minimized defects, as confirmed by comprehensive structural and spectroscopic characterizations, detailed below.

**Fig. 1.**
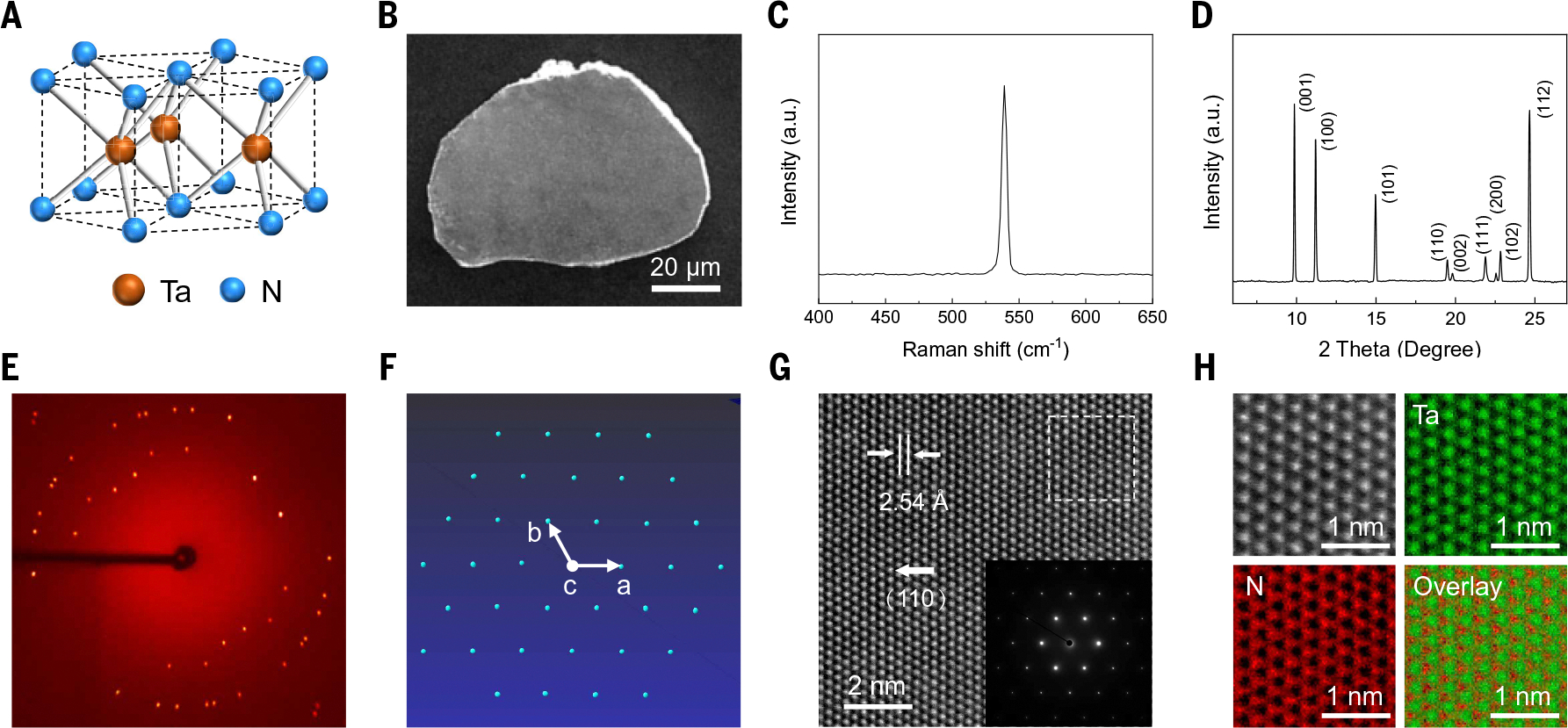
Structural characterization of single-crystalline θ-TaN. (**A**) Schematic of the hexagonal crystal structure of θ-TaN. (**B**) Scanning electron microscope image of a θ-TaN crystal. Scale bar: 20 μm. (**C**) Raman spectrum of θ-TaN crystals, confirming phase purity. a.u., arbitrary units. (**D**) X-ray diffraction (XRD) spectrum showing sharp diffraction peaks, all consistently indexed to the θ-phase. (**E**) Single-crystal XRD image of θ-TaN. (**F**) Reconstructed reciprocal lattice of θ-TaN from the complete single-crystal XRD dataset, confirming single crystallinity over the entire sample. The lattice constants were measured as *a* = *b* = 2.94 Å and *c* = 2.89 Å for θ-TaN. (**G**) HRTEM image of θ-TaN showing atomically resolved lattice planes. (Inset) Two-dimensional (2D) Fourier transform corresponding to the [001] zone axis; arrow indicates the (110) crystal direction. Scale bar: 2 nm. (**H**) Atomically resolved elemental mapping of θ-TaN lattices from EELS measurements, confirming uniform distribution of tantalum and nitrogen. EELS was performed at the boxed region in (G).

θ-TaN has a hexagonal structure in the $P\overset{-}{6}m2$ space group, where tantalum and nitrogen atoms form interpenetrating covalent networks (Fig. 1A). Scanning electron microscopy (Fig. 1B) shows the as-synthesized θ-TaN crystals, ranging from 10 to 100 μm in size, with a smooth and clean surface. The high-quality single-crystal structure of our θ-TaN samples was verified by Raman spectroscopy, single-crystal x-ray diffraction (S-XRD), high-resolution transmission electron microscopy (HRTEM), and election diffraction and electron energy-loss spectroscopy (EELS). The Raman spectroscopy data (Fig. 1C) showed a prominent peak at 544 cm^−1^, consistent with the Raman-active zone-center optical phonon mode identified in the phonon band structure of θ-phase presented in Fig. 3B from synchrotron inelastic x-ray scattering (IXS) experiments and first-principles calculations. The x-ray diffraction peaks (Fig. 1D) align precisely with the expected θ-TaN crystal planes.

Furthermore, to verify the single-crystalline nature of θ-TaN over the entire crystal, S-XRD was performed on θ-TaN samples. Under x-ray exposure, the θ-TaN sample was subjected to a full 360° rotational scan, and the diffraction data were continuously recorded and merged into a single image. Such a single crystallinity was not observed in previous θ-TaN samples because of the presence of defects and grain boundaries. In our merged plot of the S-XRD images of θ-TaN (Fig. 1E), each reflection spot appeared as a single dot without distortion, indicating that all diffraction patterns obtained through the whole crystal were consistent. This consistency confirms that the entire θ-TaN sample exhibits perfect single crystallinity with no detectable grain boundaries (42). The experimentally reconstructed reciprocal lattice from S-XRD (Fig. 1F) further confirms the crystal structure of $P\overset{-}{6}m2$ symmetry, yielding lattice constants *a* = *b* = 2.94 Å and *c* = 2.89 Å, in excellent agreement with theoretical predictions (25–27).

We used focused ion beam milling (see supplementary materials) to fabricate a θ-TaN crystal into a thin film with ~100 nm thickness for HRTEM studies. The HRTEM image (Fig. 1G) demonstrates the atomically resolved single-crystal lattice of our θ-TaN sample. The reciprocal lattice peaks measured from electron diffraction (Fig. 1G, inset) confirmed the long-range crystallographic order and were indexed with the zone axes along the [001] direction. The measured distance between each fringe is 2.54 Å, which is consistent with (110) lattice spacing of θ-TaN crystals. Furthermore, EELS spectra were acquired to enable elemental mapping of the sample. The measured EELS image (Fig. 1H) confirmed a uniform distribution of tantalum and nitrogen at the atomic scale, precisely aligned with the periodic lattice of θ-TaN.

## High thermal conductivity measured in θ-TaN

**Fig. 2.**
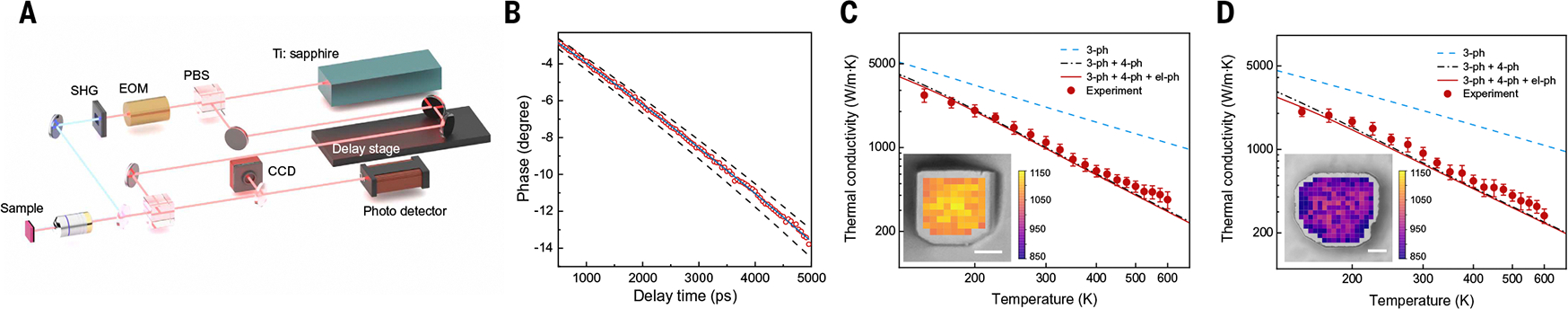
Temperature-dependent thermal conductivity measurements of θ-TaN. (**A**) Schematic of the ultrafast pump-probe setup based on TDTR. SHG, second-harmonic generator; EOM, electro-optic modulator; PBS, polarizing beam splitter; CCD, charge-coupled device camera. (**B**) Typical TDTR data: thermal reflectance phase signal versus time (red circles), fitted to the thermal transport model (blue line). Calculated curves (black dashed lines) indicate ±10% deviations in thermal conductivity to illustrate measurement accuracy. (**C** and **D**) Experimentally measured thermal conductivity of θ-TaN (red dots) in comparison to first-principles calculations considering: three-phonon scattering (blue dashed), combined three- and four-phonon scattering (black dot dashed), and full phonon and electron-phonon scattering (red solid). (Insets) 2D spatial mapping of thermal conductivity measured across the entire θ-TaN crystals. Temperature-dependent data shown along the *a* axis (C) and *c* axis (D), from 150 to 600 K. Scale bars: 20 μm.

To characterize the thermal properties and gain fundamental insight into heat conduction of θ-TaN crystals, ultrafast optical pump-probe techniques, based on the time-domain thermoreflectance (TDTR; see supplementary materials), were used to measure their thermal conductivity and study phonon transport. TDTR is a standard technique routinely used to measure thermal conductivity, including high-thermal-conductivity materials such as diamond, BAs, BP, BN, and metals (13, 14, 17, 24, 43–46). It is particularly well suited for studying θ-TaN crystals because it requires no physical contact and offers high spatial resolution at the micrometer scale. A femtosecond pump pulse induced a localized temperature rise on the sample, and a time-delayed probe pulse monitored the transient temperature decay (Fig. 2A). By measuring the full transient temperature decay as a function of time delay and fitting the data to a thermal model, the thermal conductivity was quantitatively determined. Figure 2B shows typical experimental data from our TDTR measurements and fittings. The temperature-dependent thermal conductivity of θ-TaN was measured from 150 to 600 K and for samples with both *a*-axis (Fig. 2C) and *c*-axis (Fig. 2D) crystal orientations.

We measured a room-temperature thermal conductivity of 1100 W/m·K for our θ-TaN single crystals, representing the highest value reported for any metallic materials to date. Further, we examined the sample uniformity by conducting spatially resolved thermal conductivity mapping across entire θ-TaN crystals (Fig. 2, C and D). The samples were mounted on a piezoelectric translational stage and scanned along the *x*-*y* plane. The thermal conductivity remained uniformly high across the entire crystal, measured at 1105 ± 134 W/m·K along the *a* axis and 928 ± 111 W/m·K along the *c* axis. This spatial consistency reflects the high crystallinity of the samples and confirms that the measured ultrahigh thermal conductivity originates from intrinsic lattice behavior, in strong agreement with first-principles predictions.

Furthermore, despite θ-TaN’s metallic electronic structure (fig. S4, supplementary materials), the measured temperature dependence of thermal conductivity (Fig. 2, C and D) shows a marked decline with increasing temperature, in contrast to the weak temperature dependence typically observed in conventional metals, where heat transport is dominated by electrons and limited by electron-phonon interaction (8, 9). This trend indicates that heat transport in θ-TaN is primarily phonon-mediated. Supporting this conclusion, our electrical measurements of θ-TaN (supplementary materials) yield a high electrical conductivity of ~1.5 × 10^6^ S/m, which falls within the range of typical metals (47). However, in the presence of its ultrahigh thermal conductivity, the Wiedemann-Franz law indicates that electronic contributions to heat transport are minimal. We also attribute the increase in thermal conductivity at lower temperatures to the suppression of phonon-phonon scattering, as phonon populations shift toward low-energy modes following Bose-Einstein statistics. These experimental observations are in strong agreement with first-principles calculations (Fig. 2, C and D, solid lines), confirming that θ-TaN’s ultrahigh thermal conductivity arises from long-lived phonons enabled by both extremely weak phonon-phonon interactions and electron-phonon interactions.

## IXS measurement of phonon band structure and weak phonon-phonon scattering

**Fig. 3.**
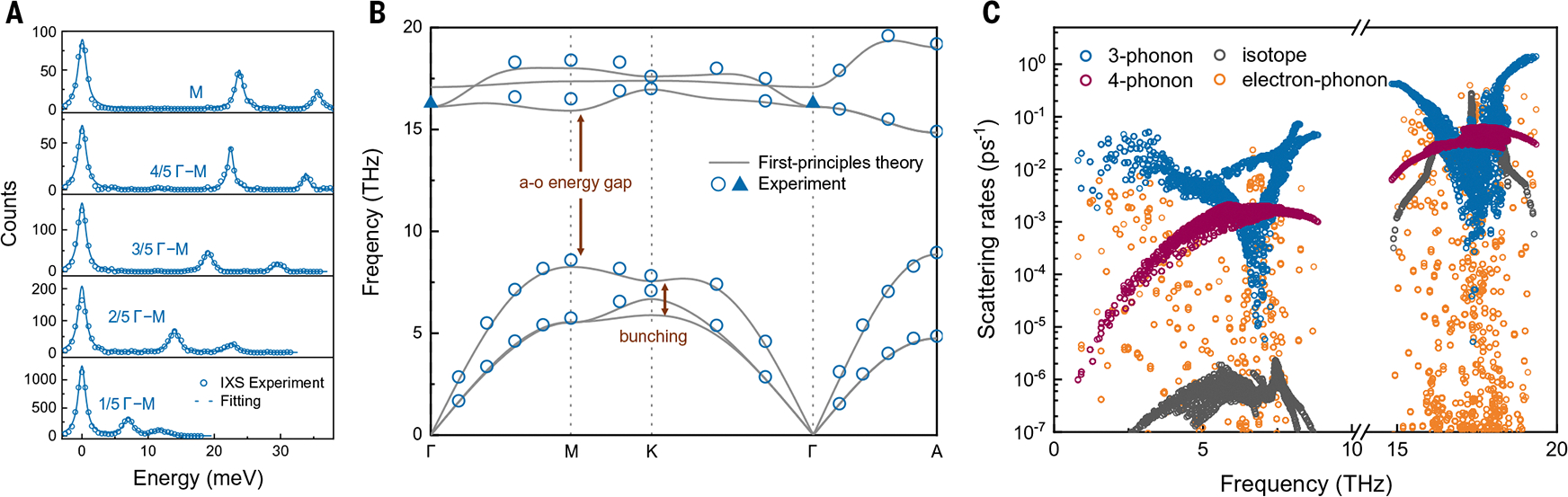
Experimental measurements and first-principles theory for phonon band structure and scattering mechanisms in θ-TaN. (**A**) Representative IXS spectrum of θ-TaN along the Γ–M direction. (**B**) Experimentally measured phonon band structure of θ-TaN from IXS (circles) and Raman spectroscopy (triangles), overlaid with first-principles calculations (solid lines). (**C**) Phonon scattering rates from first-principles calculations, showing contributions from various scattering mechanisms, including three-phonon (blue), four-phonon (purple), isotope (gray), and electron-phonon (orange) scattering processes.

The coexistence of metallic electrical conductivity and phonon-dominated thermal transport distinguishes θ-TaN from conventional metals. We directly measured the phonon band structure of θ-TaN using the IXS technique (13) (supplementary materials). Synchrotron-based x-rays were directed onto the sample and scattered inelastically from lattice vibrations, exchanging energy and momentum with phonons. The resulting IXS spectra (Fig. 3A) were acquired at various Q-points along high-symmetry paths in reciprocal space, enabling reconstruction of the phonon modes throughout the Brillouin zone.

The IXS-measured phonon band structure of θ-TaN (Fig. 3B) validated the first-principles calculations and revealed two characteristic features that influence its thermal transport. First, the IXS experiment of θ-TaN exhibited a large acoustic-optical phonon gap of ~6 THz that strongly suppressed scattering between acoustic and optical phonons. Second, a pronounced acoustic phonon bunching effect was observed in which the two transverse and one longitudinal acoustic branches remained tightly clustered in energy across the Brillouin zone. This close spacing narrowed the phase space available for phonon scattering within the acoustic branches. As dictated by energy and momentum conservation rules, these two characteristics reduced phonon-phonon scattering in θ-TaN. In addition to these intrinsic lattice dynamic features, phonon-isotope scattering in θ-TaN was exceptionally weak because of the nearly monoisotopic nature of tantalum. With the ^181^Ta isotope making up 99.988% of natural tantalum, mass disorder–induced scattering was effectively eliminated. Isotope scattering had only a negligible contribution to phonon dissipation and preserved long phonon lifetimes.

To quantitatively assess the contributions of different scattering mechanisms (7), phonon scattering rates calculated from first-principles theory were plotted with decomposition into four main channels: three-phonon, four-phonon, isotope, and electron-phonon interactions. As shown in Fig. 3C, among these, three-phonon processes dominated across most of the phonon spectrum but were suppressed in the high-frequency acoustic range (6 to 8 THz) because of the combined effects of the phonon bandgap and acoustic bunching. This suppression amplified the relative contribution of higher-order four-phonon scattering, which is typically negligible in most materials where thermal transport is governed only by three-phonon scattering (11, 13, 25). Also, as expected for natural isotopic abundance, isotope scattering remains minimal across the entire phonon spectrum. Although electron-phonon scattering is typically a major limiting factor for thermal conductivity in metallic materials, θ-TaN represents an exception wherein such scattering contributed negligibly to the overall phonon scattering rates.

## Weak electron-phonon interactions and ultrafast dynamics in θ-TaN

**Fig. 4.**
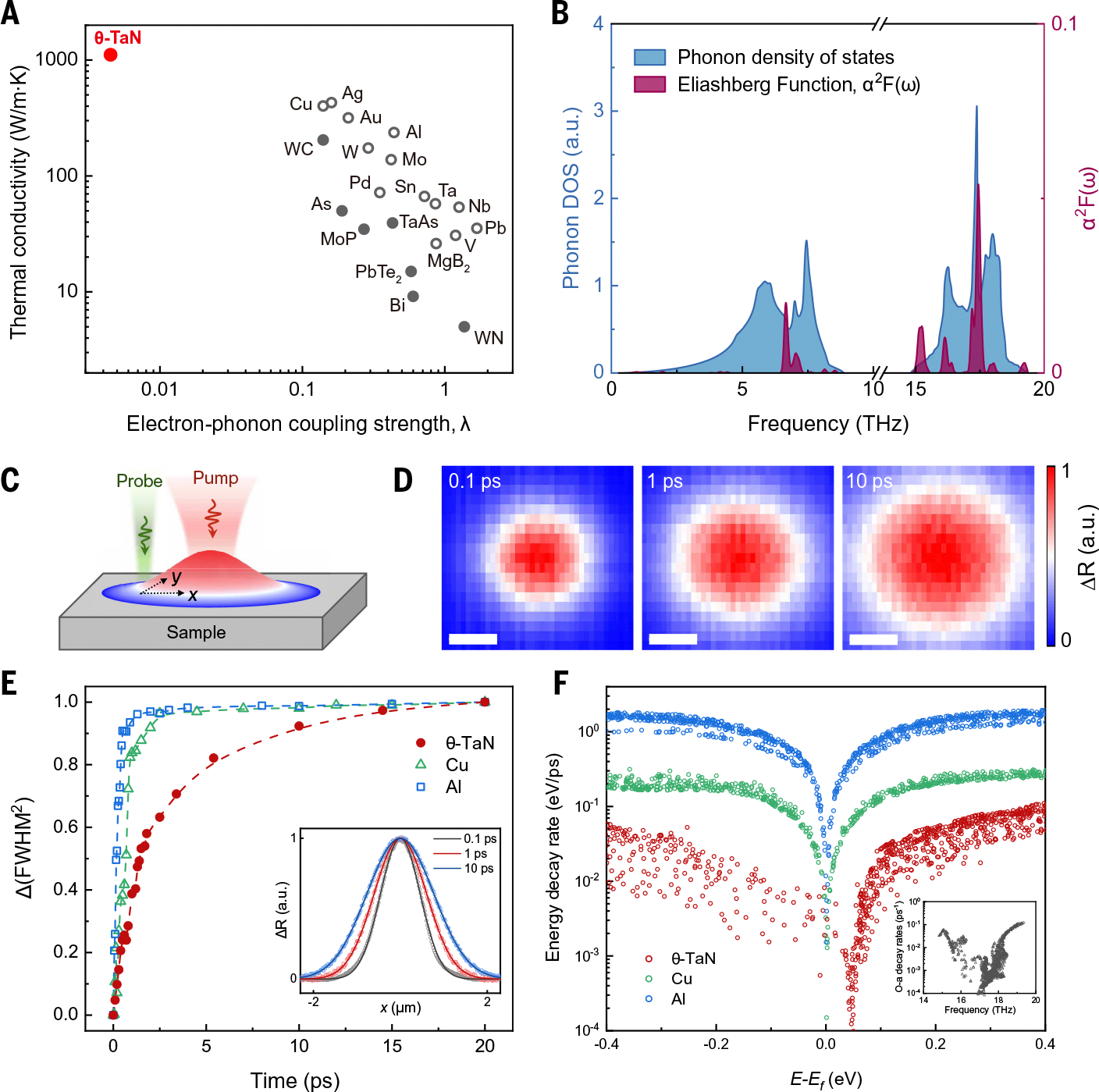
Experimental measurements and first-principles calculations on electron-phonon interactions and ultrafast dynamics in θ-TaN. (**A**) Summary of different metallic materials, plotted by thermal conductivity (50–56) versus electron-phonon coupling strength (λ) (57–66), highlighting θ-TaN as a new benchmark with exceptionally high thermal conductivity and weak coupling. (**B**) Mode-resolved phonon density of states and Eliashberg function α^2^*F*(ω), showing much weaker electron-phonon coupling in the acoustic versus optical phonon range. (**C**) Schematic of the transient reflection spectroscopy experiment for characterizing carrier relaxation dynamics through ultrafast pump-probe measurements. (**D**) The measurement data for time-resolved 2D mapping of photoexcited carrier diffusion in θ-TaN. Scale bars: 1 μm. (**E**) Time-dependent electron relaxation profiles measured for θ-TaN (red), Cu (green), and Al (blue), quantified by the squared full width at half maximum (FWHM^2^) of Gaussian fits (inset). The relaxation time in θ-TaN is measured to be ~15 ps, an order of magnitude longer than that of Cu or Al, indicating very weak electron-phonon interactions. (**F**) First-principles calculations of the mode-resolved electron energy decay rate through electron-acoustic phonon scattering in θ-TaN (red), Cu (green), and Al (blue). (Inset) Optical-to-acoustic phonon decay rates in θ-TaN.

We compared all metallic materials on the basis of their electron-phonon coupling strength and corresponding thermal conductivity (Fig. 4A). In most metals and semimetals, electron-phonon interactions are strong and contribute to phonon scattering, thereby limiting lattice thermal conductivity. In contrast, θ-TaN exhibited the weakest electron-phonon coupling strength (λ ≈ 0.0045 at 300 K), making it a distinct metallic material as characterized by phonon-dominated heat transport.

To understand the microscopic origin of this weak coupling, we performed first-principles calculations of the Eliashberg spectral function $\alpha^{2}F(\omega )$, defined as

(1) $$\alpha^{2}F(\omega )=\frac{1}{2}\sum_{\nu }\int_{BZ}\omega_{q\nu }\lambda_{q\nu }\delta \left(\omega -\omega_{q\nu }\right)\frac{dq}{\Omega_{BZ}}$$

where $\omega_{q\nu }$ is the phonon frequency, $\lambda_{q_{\nu }}$ is the mode-resolved electron-phonon coupling strength, $\Omega_{BZ}$ is the Brillouin zone volume, and δ is the Dirac delta function. Physically, $\alpha^{2}F(\omega )$ is the key parameter that quantifies a phonon frequency–resolved measure of electron-phonon scattering, reflecting the phonon density of states weighted by each mode’s coupling strength to electronic states at the Fermi level (48). The profile of $\alpha^{2}F(\omega )$ pinpoints which phonon modes couple most effectively with electrons.

As shown in Fig. 4B, $\alpha^{2}F(\omega )$ remained low across the entire phonon spectrum and was especially weak in the acoustic phonon regime (<8 THz), where the dominant heat-carrying modes reside. Modest increases appeared in higher-frequency optical modes (15 to 20 THz), indicating limited electron-optical phonon coupling. The result was consistent with theoretical analyses of direct *d-d* bonding and electronic density of states (25).These calculations indicated very weak electron-phonon coupling that preserved the high thermal conductivity and supported efficient phonon-mediated heat transport in θ-TaN.

To experimentally verify such weak electron-phonon coupling in θ-TaN, we conducted ultrafast pump-probe spectroscopy based on transient reflectivity microscopy to directly measure electron relaxation dynamics (supplementary materials). A femtosecond pump pulse excited electrons above the Fermi level, generating a population of hot electrons (Fig. 4C). These electrons subsequently relaxed by dissipating energy to the lattice through electron-phonon interactions and diffused away from the excitation region as a result of concentration gradients. A time-delayed probe pulse monitors transient changes in reflectivity (Δ*R*), capturing the time evolution of the hot electron diffusion and energy relaxation. By scanning the probe beam across the pump spot with a galvanometer mirror, the system enables space-and time-resolved mapping of the relaxation process.

Maps of the spatial profiles of electron diffusion at various time delays for θ-TaN are shown in Fig. 4D. The diffusion length is represented by the full width at half maximum (FWHM) of the spatial Δ*R* profiles (Fig. 4E, inset), and the time evolution of Δ(FWHM^2^) reflects the dynamics of electron energy relaxation to the lattice (Fig. 4E). In representative metals such as Cu and Al, relaxation times are ~1 ps, consistent with their relatively strong electron-phonon coupling. In contrast, θ-TaN is measured with a markedly prolonged relaxation time of ~15 ps (Fig. 4E), providing direct experimental evidence of its exceptionally weak electron-phonon interactions.

Electron relaxation dynamics can occur directly through electron-acoustic phonon interactions and indirectly through electron-optical phonon coupling followed by thermalization through optical-to-acoustic phonon scattering, with acoustic phonons serving as the primary heat carriers. In θ-TaN, the former pathway is intrinsically suppressed given the weak electron-acoustic phonon coupling, as indicated by the Eliashberg spectral function in Fig. 4B. The indirect pathway is likewise limited by the large phonon bandgap (Fig. 3B), which restricts the phase space for optical phonon decay into acoustic modes.

To quantify these processes, we performed first-principles, mode-resolved calculations for hot electron energy decay (49). As shown in Fig. 4F, near the Fermi level, energy decay rate through the direct electron-acoustic phonon channel in θ-TaN was more than an order of magnitude weaker than in Cu or Al (Fig. 4F), consistent with our ultrafast experiment and resulting in relaxation times on the order of ~15 ps. The indirect channel was similarly constrained, with optical phonon decay rates mostly remaining below 0.1 ps^−1^ (Fig. 4F, inset). These dual bottlenecks delayed hot electron thermalization and explained the extended relaxation time measured in θ-TaN.

## Conclusions

We measured an ultrahigh thermal conductivity of ~1100 W/m·K at room temperature in single-crystal θ-TaN. Our study experimentally verified the first-principles theory and establishes θ-TaN as a benchmark for high-thermal-conductivity metallic materials. Comprehensive thermal and structural measurements, combined with IXS to determine phonon band structure, revealed the microscopic origins of this exceptional behavior. The large acoustic-optical phonon gap, strong acoustic phonon bunching, and minimal isotope scattering collectively suppressed phonon-phonon interactions. Ultrafast pump-probe spectroscopy further confirmed exceptionally weak electron-phonon coupling and phonon-dominated heat transport, distinguishing it from conventional metals. These findings redefine the upper limit of thermal conductivity in metallic materials and introduce new design principles for engineering high-performance thermal management. The ultrahigh thermal conductivity of θ-TaN, together with its metallic nature, and manufacturing integration could revolutionize the future technology paradigms for thermal management in advanced electronics, aerospace, energy, and power systems.

## Supplementary Material

Supplementary Materials

science.org/doi/10.1126/science.aeb1142

Materials and Methods; Figs. S1 to S4; References (67–98)

## Data Availability

All data and details of materials synthesis are available in the main text or the supplementary materials.
