# Supplementary material for: Metallic θ-phase tantalum nitride has a thermal conductivity triple that of copper
Source: Science. Author manuscript; Available in PMC 2026 Jul 15. (PMC13371953; doi:10.1126/science.aeb1142)
Supplement: Supplementary Materials [file NIHMS2185822-supplement-Supplementary_Materials.pdf]

Science AAAS

Supplementary Materials for

Metallic θ-phase tantalum nitride has a thermal conductivity triple that of copper

Suixuan Li *et al*.

Corresponding author: Yongjie Hu, yhu@seas.ucla.edu

Science **391**, 707 (2026)
DOI: 10.1126/science.aeb1142

The PDF file includes:

Materials and Methods
Figs. S1 to S4
References

Materials and Methods

Material synthesis and sample preparation

Theta-tantalum nitride (θ-TaN) crystals were synthesized using a modified synthesis method based on a previous metathesis reaction (*67*). Inside an Ar-filled glovebox (O_2, $H_2O \leq 1$ ppm), Ta_2O_5 powder (99.9%, FUJIFILM Wako, 0.41 mmol), NaN_3 powder (98%, FUJIFILM Wako, 0.59 mmol), and Na metal (99.95%, Nippon Soda, 4.2 mmol) were placed into a BN crucible (99.5%, Showa Denko). After loading the reaction materials, the crucible was sealed inside a stainless-steel (SUS316) container and heated to 1373 K. The system was maintained at this temperature for 10 hours and then furnace-cooled to room temperature. Following synthesis, the crucible was removed from the container. The product was washed sequentially with denatured ethanol (86% ethanol, 14% isopropanol) and distilled water, and then dried in air at 353 K.

Structural Characterizations

Transmission electron microscopy and energy-loss electron spectroscopy characterizations

The θ-TaN crystal sample for high-resolution transmission electron microscope (TEM) imaging was prepared using a focused ion beam (FIB) system (Nova 600, FEI). First, a single-crystal specimen was cut into a small slab measuring 8 μm × 8 μm × 2 μm (width × height × thickness) using the FIB. A nanomanipulator was then used to transfer the slab onto a TEM sample holder (PELCO FIB Lift-Out, Ted Pella). The slab was further thinned using the FIB and subsequently refined with a concentrated argon ion beam (Model 1040 NanoMill, Fischione) until its thickness was reduced to below 100 nm, allowing for effective electron beam penetration during TEM imaging. Once milling was complete, the sample was transferred to a high-angle annular dark-field (HAADF) aberration-corrected scanning TEM instrument (Grand ARM, JEOL) for TEM imaging and electron diffraction. Electron Energy Loss Spectroscopy (EELS) was performed to characterize the chemical composition of the sample through measurements of electron energy loss during sample interaction. The images were processed using Gatan TEM software.

X-ray diffraction (XRD) measurements

Single-crystal X-ray diffraction (SXRD) analysis was conducted using a Bruker SMART APEX II system equipped with a CCD detector at the UCLA X-ray crystallography facility. A θ-TaN single crystal (~80 μm size) was mounted onto the goniometer stage for measurement. To

verify the crystal's single crystallinity, the sample was subjected to a full 360-degree rotational scan under X-ray exposure, with diffraction data continuously recorded and compiled into a single image. Subsequently, to determine the unit cell structure and identify any potential twinning or structural defects, high-resolution XRD measurements were performed using a Mo Kα radiation source. The measurements employed a frame width of 0.3 degrees and an exposure time of 60 seconds per frame. The collected diffraction data were analyzed using Bruker APEX software, revealing a hexagonal crystal structure in the P-6m2 space group, with lattice constants determined as $a = b = 2.94$ Å and $c = 2.89$ Å. In addition, powder X-ray diffraction was conducted at Beamline 12.2.2 of the Advanced Light Source (ALS), Lawrence Berkeley National Laboratory (*68*), which measured the same lattice constants. Reciprocal lattice (k-space) mapping was constructed from the single-crystal diffraction data to visualize the crystal's structural integrity, confirming precise crystallographic alignment. The comprehensive analysis demonstrated that the θ-TaN crystal was a high-quality, homogeneous single-crystal domain, free of impurities, twinning, or structural defects.

Raman spectroscopy

Raman spectroscopy was performed using a confocal micro-Raman system (inVia, Renishaw) with a 633 nm laser excitation and a 1200 mm^{-1} grating. Measurements were performed in a backscattering geometry with a polarized laser, integrated with a Leica DM2500 optical microscope. A 50×/0.75 objective lens provided a lateral spatial resolution of approximately 0.5 μm.

Inelastic X-ray scattering measurement

Inelastic X-ray scattering (IXS) experiments were conducted at Sector 30 of the Advanced Photon Source (APS), Argonne National Laboratory. The incident synchrotron X-ray beam had an energy of 23.71 keV, with an energy resolution of 1.5 meV. The focused X-ray spot size on the sample was approximately 35 μm × 10 μm. The scattered X-ray are detected with transmission mode. The orientation matrix of θ-TaN single crystals was first determined via X-ray diffraction. Phonon dispersion was then measured by aligning various crystallographic orientations to access high-symmetry directions across the Brillouin zone. IXS spectra were collected over an energy transfer range from –5 meV to 90 meV, capturing both acoustic and optical phonon modes (*13*, *69*, *70*).

Thermal and Charge Carrier Transport Characterizations

Time-domain thermoreflectance principles

The time-domain thermoreflectance (TDTR) is an established technique for measuring thermal conductivity and has been applied to a broad range of materials, including high thermal conductivity materials such as diamond, boron nitride, boron phosphide, copper, nickel, tungsten, and various metals, as well as the recent discovery of high thermal conductivity in boron arsenide and isotope-enriched boron nitride (*13–17*, *24*, *43–46*, *71–82*). In the TDTR setup, a Ti:Sapphire oscillator (Tsunami, Spectra-Physics) was used to generate femtosecond laser pulses (~100 fs) at a repetition rate of 80.7 MHz and a central wavelength at 800 nm. The laser output was split into pump and probe beams. The pump beam was modulated by an electro-optic modulator (EOM) and frequency-doubled to 400 nm via second harmonic generation in a bismuth triborate (BIBO) crystal. The modulated pump pulse induced a rapid temperature rise at the sample surface. The transient temperature decay was then tracked by the time-delayed probe beam via a lock-in amplifier, using a mechanical delay stage to achieve sub-picosecond time resolution. Thermal conductivity was determined by fitting the measured thermoreflectance decay to a multilayer thermal model. The thermal modeling methods are summarized below; additional details on the TDTR experiments and modeling are available in the previous literature publications from us (*13*, *14*, *17*, *24*, *43–46*, *75*, *76*) and others (*15*, *16*, *71–74*, *77–82*).

The energy contained in each pump laser pulse, with amplitude Q, is sampled by modulation, yielding the heat input $Q(\omega)$:

$$Q(\omega) = \frac{2\pi Q}{T} \sum_{k=-\infty}^{\infty} \delta(\omega - \omega_0 - k\omega_s)\ e^{-ik\omega_0 T_0} \tag{1}$$

where ω_0 is the EOM modulation frequency, ω_s is the sampling frequency, and T is the pulse period. To characterize the thermal response of the substrate, the temperature and heat flux at the top and bottom surfaces are related through matrix equations for bulk layers (Equation 2) and interfaces (Equation 3):

$$\begin{bmatrix} \theta_b \\ f_b \end{bmatrix} = \begin{bmatrix} \cosh(qd) & \frac{-1}{\kappa_z q}\sinh(qd) \\ -\sigma_z q \sinh(qd) & \cosh(qd) \end{bmatrix} \begin{bmatrix} \theta_t \\ f_t \end{bmatrix} \tag{2}$$

$$\begin{bmatrix} \theta_b \\ f_b \end{bmatrix} = \begin{bmatrix} 1 & G^{-1} \\ 0 & 1 \end{bmatrix} \begin{bmatrix} \theta_t \\ f_t \end{bmatrix} \tag{3}$$

where θ_b and θ_t are the top and bottom temperatures, f_b and f_t are the top and bottom heat fluxes, κ_z is the cross-plane thermal conductivity, d is the slab thickness and $q^2 = \frac{iw}{\alpha}$, with α as the thermal diffusivity. Mathematically, for the multilayer structure, the temperature and heat flux correlation follows:

$$\begin{bmatrix} \theta_b \\ f_b \end{bmatrix} = M_n M_{n-1} \dots M_1 = \begin{bmatrix} A & B \\ C & D \end{bmatrix} \begin{bmatrix} \theta_t \\ f_t \end{bmatrix} \tag{4}$$

The unit thermal response $H(\omega)$ is then given as:

$$H(\omega) = \frac{A_0}{2\pi} \int_0^{\infty} k \left(\frac{-D}{C}\right) \left(\frac{A_0}{2\pi}\right) \exp\left(\frac{-k^2(w_0^2 + w_1^2)}{8}\right) dk \tag{5}$$

where A_0 is the absorbed energy per unit pump laser, and w_0, w_1 are the pump and probe beam radii. With the thermal response under pump laser give as $\Theta(\omega) = H(\omega)Q(\omega)$ and by applying the sampling theorem to extract the frequency component, the transfer function obtained from reflected probe signal is:

$$Z(\omega_0) = \frac{\beta Q Q_{\text{probe}}}{T^2} \sum_{k=-\infty}^{\infty} H(\omega_0 + k\omega_s)\, e^{ik\omega_s \tau} \tag{6}$$

The lock-in amplifier picks up in-phase an out-of-phase components of the transfer function, i.e., $X = \mathrm{Re}\{Z(\omega_0)\}$ and $Y = \mathrm{Im}\{Z(\omega_0)\}$, and the amplitude and phase signal are obtained as $\sqrt{X^2 + Y^2}$ and the arctangent value of Y/X, respectively.

Sample preparation and TDTR measurement details

The synthesized θ-TaN crystals exhibited nanoscale-smooth surfaces and were transferred onto a silicon wafer for thermal measurements, following procedures similar to our prior sample preparation for high-thermal-conductivity crystals such as BAs and BP (*13*, *14*, *44*). A thin aluminum transducer layer was deposited using an electron beam evaporator (CHA Mark 30), and its thickness was determined by atomic force microscopy and picosecond ultrasonics (*83*).

Consistent with prior TDTR studies on BAs and BP (*14*, *44*), no appreciable steady-state heating was observed. The steady-state temperature rise was analyzed following the literature (*46*, *71*, *72*, *84*, *85*). In the low-frequency limit, the temperature rise is given by

$$\Delta T_0 = \frac{A_0}{2\sqrt{\pi} w_0 \kappa} \quad (7)$$

where A_0 is the absorbed pump power in the transducer, w_0 is the pump-beam radius, and κ is the thermal conductivity. Under our experimental conditions, the TDTR measurements produced negligible heat accumulation (< 1 K).

During the TDTR experiment, laser spot sizes and modulation frequencies were systematically varied for the measurements (Figure S2 and Figure S3).

Heat capacity of θ-TaN

The temperature-dependent volumetric heat capacity of θ-TaN is shown in Figure S1. The heat capacity per unit cell was determined from first-principles lattice dynamics calculations. The crystal structure and lattice parameters were further experimentally verified with single-crystal x-ray diffraction and TEM studies, as described in the main text and in other sections of this Supplementary Materials.

Thermal penetration depth

TDTR is a transient technique and the characteristic heat transfer length is evaluated by the thermal penetration depth (*5*),

$$\delta_T(T,f) = \sqrt{\frac{\kappa}{\pi\, C\, f}} \qquad (8)$$

Where f is the modulation frequency and C is the volumetric heat capacity. For modulation frequencies between 3 and 10 MHz, δ_T is calculated to be 3.5–6.4 μm.

Electronic band structure and metallic nature of θ-TaN

Figure S4 displays the electronic band structure of θ-TaN, calculated with fully relativistic ONCV pseudopotentials including spin-orbit coupling. Multiple bands, primarily from Ta-*d* and N-*p* orbitals, cross the Fermi level (E_f) along the Γ–K–M and Γ–A directions, with no direct or indirect gap opens anywhere in the Brillouin zone. The electronic band structure confirms the intrinsically metallic nature of θ-TaN. The electronic bands and metallic nature of θ-TaN are consistent with recent reports in literature (*25–27*).

Electrical transport measurement of θ-TaN

The electrical conductivity of θ-TaN was determined using the van der Pauw technique on single-crystal samples. Four ohmic contacts were affixed at the sample perimeter. A constant current was applied between two adjacent contacts, and the corresponding transverse voltage was measured across the remaining contact pair. The electrical resistance was determined following van der Pauw formula. To reduce systematic errors from geometric asymmetry and contact misalignment, measurements were performed in all four current-voltage configurations and averaged. Finally, the electrical conductivity, σ, was calculated by converting the measured resistance, R_s, using the relationship $\sigma = 1/(R_s \cdot t)$, where t is the sample thickness precisely measured by profilometry. The electrical conductivity is measured to be $\sim 1.5 \times 10^6$ S/m, which is well within the range of metallic materials (*47*) and confirms the metallic nature of θ-TaN.

Ultrafast electron dynamics measurement

Transient reflectivity microscopy (TRM) is a well-established ultrafast pump–probe technique for directly probing electron–phonon coupling and carrier dynamics in materials (*86–88*). In the TRM setup, a femtosecond pump pulse is focused onto the θ-TaN surface using a 50× Mitutoyo objective to generate a localized excess carrier distribution. The laser wavelengths and

fluences were characterized to maintain a linear reflectance response. Spatial diffusion dynamics of photoexcited carriers are imaged using a galvanometer mirror to scan the delayed probe beam across the sample. Temporal evolution is recorded through systematic delay scanning of the probe pulse, enabling sub-picosecond resolution.

At each delay, the spatial profile of the TRM signal at each delay is fit to a Gaussian function (*86–88*):

$$\Delta R(x, y) = A \exp\left[-\frac{4ln2 \cdot [(x - w_0)^2 + (y - w_1)^2]}{\mathrm{FWHM}^2}\right] \tag{9}$$

where w_0 and w_1 represent the center position and FWHM defines the carrier cloud width. Carrier diffusion is quantified via the mean-square expansion of FWHM over time (*86–88*):

$$D = \frac{1}{16ln2} \cdot (\mathrm{FWHM}_t^2 - \mathrm{FWHM}_0^2)/t \tag{10}$$

where *D* is the diffusion coefficient and t is the time delay. Crucially, the time-dependent diffusion coefficient reflects the relaxation of hot electrons, which is dominated by electron–phonon scattering (*86–88*). The decay of *D(t)* thus provides a direct probe of electron-phonon interaction strength and dynamics, enabling quantitative analysis of coupling rates.

First-Principles Calculations

First-principles theory and calculations for lattice dynamics

The first-principles calculations are following the settings in recent literature (*22*, *23*, *25–27*, *89*).

The vibrational dynamics of a crystal lattice arise from the interatomic potential, which can be expressed as a Taylor series expansion in terms of atomic displacements:

$$\begin{aligned} U = U_0 &+ \frac{1}{2} \sum_{\{l,b,\mu\}} \Phi_{\mu_1\mu_2}(\boldsymbol{l}_1\boldsymbol{b}_1; \boldsymbol{l}_2\boldsymbol{b}_2) u_{\mu_1}(\boldsymbol{l}_1\boldsymbol{b}_1) u_{\mu_2}(\boldsymbol{l}_2\boldsymbol{b}_2) \\ &+ \frac{1}{3!} \sum_{\{l,b,\mu\}} \Phi_{\mu_1\mu_2\mu_3}(\boldsymbol{l}_1\boldsymbol{b}_1; \boldsymbol{l}_2\boldsymbol{b}_2; \boldsymbol{l}_3\boldsymbol{b}_3) u_{\mu_1}(\boldsymbol{l}_1\boldsymbol{b}_1) u_{\mu_2}(\boldsymbol{l}_2\boldsymbol{b}_2) u_{\mu_3}(\boldsymbol{l}_3\boldsymbol{b}_3) \\ &+ \frac{1}{4!} \sum_{\{l,b,\mu\}} \Phi_{\mu_1\mu_2\mu_3\mu_4}(\boldsymbol{l}_1\boldsymbol{b}_1; \boldsymbol{l}_2\boldsymbol{b}_2; \boldsymbol{l}_3\boldsymbol{b}_3; \boldsymbol{l}_4\boldsymbol{b}_4) u_{\mu_1}(\boldsymbol{l}_1\boldsymbol{b}_1) u_{\mu_2}(\boldsymbol{l}_2\boldsymbol{b}_2) u_{\mu_3}(\boldsymbol{l}_3\boldsymbol{b}_3) u_{\mu_4}(\boldsymbol{l}_4\boldsymbol{b}_4) \\ &+ \cdots \end{aligned} \tag{11}$$

where U_0 is the equilibrium potential, and the summation is performed over all indexed cells $\boldsymbol{l}$, atoms $\boldsymbol{b}$, and direction μ, with $\Phi_{\mu_1,\dots,\mu_n}(\boldsymbol{l}_1\boldsymbol{b}_1;\dots;\boldsymbol{l}_n\boldsymbol{b}_n)$ representing the nth-order interatomic force constant (IFC) and $u_\mu(\boldsymbol{lb})$ denoting the atomic displacement of the corresponding atom in the specified direction.

Using the 2nd-order IFCs, phonon band structure can be solved for any given phonon wavevector $\mathbf{q}$ through diagonalizing the dynamical matrix,

$$D_{\mu_1\mu_2}(\boldsymbol{b}_1\boldsymbol{b}_2|\mathbf{q}) = \frac{1}{\sqrt{m_{\boldsymbol{b}_1}m_{\boldsymbol{b}_2}}}\Phi^*_{\mu_1\mu_2}(\boldsymbol{b}_1\boldsymbol{b}_2|\mathbf{q}) \tag{12}$$

such that $\sum_{\boldsymbol{b}_2,\mu_2} D_{\mu_1\mu_2}(\boldsymbol{b}_1\boldsymbol{b}_2|\mathbf{q})e_{\mu_2}(\boldsymbol{b}_2|\mathbf{q}s) = \omega^2(\mathbf{q}s)e_{\mu_1}(\boldsymbol{b}_1|\mathbf{q}s)$, with e being the phonon eigenvector corresponding to the phonon branch index s. Phonon group velocity $\boldsymbol{v}_{\mathbf{q}s}$ is further extracted from the derivative of the dynamical matrix with respect to $\mathbf{q}$. Note that $\Phi_{\mu_1\cdots\mu_{n-1}}$ is the Fourier transform of the nth-order IFCs:

$$\Phi_{\mu_1\cdots\mu_{n-1}}(\boldsymbol{b}_1\cdots\boldsymbol{b}_n|\mathbf{q}_1\cdots\mathbf{q}_{n-1}) = \sum_{\boldsymbol{h}_1\cdots\boldsymbol{h}_{n-1}}\Phi_{\mu_1\cdots\mu_{n-1}}(\mathbf{0}\boldsymbol{b}_1;\boldsymbol{h}_1\boldsymbol{b}_2;\cdots;\boldsymbol{h}_{n-1}\boldsymbol{b}_n)e^{i(\mathbf{q}_1\cdot\boldsymbol{h}_1+\cdots\mathbf{q}_{n-1}\cdot\boldsymbol{h}_{n-1})} \tag{12}$$

where we define $\boldsymbol{h}_i = \boldsymbol{b}_{i+1} - \boldsymbol{b}_1, \forall i$.

With the phonon band structure in the first Brillouin zone obtained through diagonalizing the dynamic matrices for each wavevector $\mathbf{q}$, the volumetric heat capacity is evaluated as,

$$C_V(T) = \frac{1}{V}\sum_{\nu}\int_{\text{BZ}}\hbar\omega_{\mathbf{q}\nu}\frac{\partial}{\partial T}\left[\left(\exp\left(\frac{\hbar\omega_{\mathbf{q}\nu}}{k_{\text{B}}T}\right)-1\right)^{-1}\right]\frac{d\mathbf{q}}{\Omega_{\text{BZ}}} \tag{13}$$

As a result, the calculated volumetric heat capacity is plotted in Figure S1.

Using IFCs calculated up to the 4th-order, phonon-phonon interactions can be determined by the quantum perturbation theory, resulting in the steady-state phonon Boltzmann transport equation (BTE):

$$\boldsymbol{v}_{\mathbf{q}s}\cdot\nabla T\frac{\partial n_{\mathbf{q}s}}{\partial T} = \left(\frac{\partial n_{\mathbf{q}s}}{\partial t}\right)_{\text{scatt}} = \left(\frac{\partial n_{\mathbf{q}s}}{\partial t}\right)_{\text{3ph}} + \left(\frac{\partial n_{\mathbf{q}s}}{\partial t}\right)_{\text{4ph}} + \left(\frac{\partial n_{\mathbf{q}s}}{\partial t}\right)_{\text{iso}} + \left(\frac{\partial n_{\mathbf{q}s}}{\partial t}\right)_{\text{el}} \tag{14}$$

in which the phonon scattering term is treated as the summation of different phonon scattering mechanisms including three-phonon scattering, four-phonon scattering, isotope scattering, as well as phonon-electron scattering. The calculation of phonon-phonon and phonon-isotope scattering matrix elements is reported in detail in previous work and the determination of the phonon-electron scattering is summarized below (*22*, *23*, *25–27*, *89*).

The deviation from equilibrium is considered as $\delta n_{\mathbf{q}s} = n_{\mathbf{q}s} - n_{\mathbf{q}s}^{\text{eq}}$, gives linearized phonon BTE as:

$$\boldsymbol{v}_{\mathbf{q}s} \cdot \nabla T \frac{\partial n_{\mathbf{q}s}^{\text{eq}}}{\partial T} = \sum_{\mathbf{q}'s'} \left(A_{\text{3ph}} + A_{\text{4ph}} + A_{\text{iso}} + A_{\text{el}}\right)_{\mathbf{q}s,\mathbf{q}'s'} \delta n_{\mathbf{q}'s'} \tag{15}$$

with the elements of matrices A_{3ph}, A_{4ph}, A_{iso}, and A_{el} quantifying the transition rates from state $\mathbf{q}'s'$ to $\mathbf{q}s$ due to corresponding scattering mechanisms. With the temperature gradient applied at given direction β, heat flux along direction α is thus $\text{J}^{\alpha} = \sum_s \langle \hbar\omega v^{\alpha} \delta n^{\beta} \rangle_{\mathbf{q}s} = -\kappa^{\alpha\beta} \nabla_{\beta} T$ with $\delta n_{\mathbf{q}s}^{\beta}$ being the exact solution of the linearized BTE under the temperature gradient $\nabla_{\beta} T$. Here, we use the bracket $\langle \phi \rangle_{\text{BZ}} = \int_{\text{BZ}} \phi \frac{d\mathbf{q}}{\Omega_{\text{BZ}}}$ to denote the integration of an arbitrary function ϕ within the Brillouin zone. Through this formulation, anisotropic thermal conductivity $\kappa^{\alpha\beta}$ can be solved iteratively until convergence.

To perform the calculation, an irreducible displacement set based on a 250-atom supercell in real space was generated. With the cutoffs for 2nd-, 3rd-, and 4th-order IFCs being configured as infinity, 8th-order neighboring atoms, and 3rd-order neighboring atoms, respectively, 1,664 displacement patterns were generated with displacement magnitudes as 0.01 Å, 0.03 Å, and 0.05 Å. For each displaced configuration, the interatomic forces were calculated via DFT using Quantum ESPRESSO (QE) (*90*) with the projector-augmented wave approach (*91*). Perdew-Zunger parameterization (*92*) of the local density approximation was employed for the exchange and correlation components in DFT. On this basis, the ALAMODE package (*93*) was used to extract the IFCs by fitting the obtained displacement-force dataset using least-square method.

The scattering matrix was constructed and the phonon BTE was solved. The tetrahedron scheme (*94*) is applied for the integration over the Brillouin zone, leading to the convergence of the solution at a relatively lower mesh density compared with smearing method. The **q**-mesh for solving the phonon BTE is 18 × 18 × 18 with ensured convergence in thermal conductivity.

Determination of electron-phonon coupling

The electron-phonon coupling strength is captured by the 1st-order electron-phonon matrix with the element for electron-phonon triplet to describe the interaction of $\mathbf{q}s + n\mathbf{k} \leftrightarrow m(\mathbf{k}+\mathbf{q})$:

$$g_{mn,s}(\mathbf{k},\mathbf{q}) = \langle \psi_{m\mathbf{k}+\mathbf{q}} | \partial_{\mathbf{q}s} V | \psi_{n\mathbf{k}} \rangle \tag{16}$$

Here, $\mathbf{q}s$ is the phonon mode, $\mathbf{k}$ and $\mathbf{k}+\mathbf{q}$ denote electron wavevectors and n, m are the electron band indices. ψ is the electron wavefunction, and ∂V is the perturbing potential associated with specific phonon mode. The magnitude of the complex value of $g_{mn,s}(\mathbf{k},\mathbf{q})$ measures the strength of such electron-phonon triplet. For a specific phonon mode $\boldsymbol{q}s$, the total electron-phonon coupling strength $\lambda_{\mathbf{q}s}$ is given by the integration over all the involved triplets normalized by the electron density of states per spin at Fermi level N_{F}:

$$\lambda_{\mathbf{q}s} = \frac{2}{N_{\mathrm{F}}\omega_{\mathbf{q}s}} \sum_{mn} \langle |g_{mn,s}(\mathbf{k},\mathbf{q})|^2 \delta(\varepsilon_{n\mathbf{k}} - \varepsilon_{\mathrm{F}}) \delta(\varepsilon_{m\mathbf{k}+\mathbf{q}} - \varepsilon_{\mathrm{F}}) \rangle_{\mathrm{BZ}} \tag{17}$$

where ε is the electron energy at certain band and wavevector, ε_{F} is the Fermi energy. The total electron-phonon coupling strength is thus given by the average of all phonon modes, $\lambda = \sum_s \langle \lambda_{\mathbf{q}s} \rangle$. The Eliashberg spectral function $\alpha^2 F$ is given by the mode-resolved coupling strength $\lambda_{\mathbf{q}s}$ and the corresponding phonon frequency, that is,

$$\alpha^2 F(\omega) = \sum_s \langle \omega_{\mathbf{q}s} \lambda_{\mathbf{q}s} \delta(\omega - \omega_{\mathbf{q}s}) \rangle_{\mathrm{BZ}} \tag{18}$$

Each phonon-electron scattering event involves two electrons and one phonon, therefore, the aforementioned scattering matrix A_{el} only have diagonal terms. For mode $\mathbf{q}s$, the diagonal term is the negative of the scattering rate, i.e., $(A_{\mathrm{el}})_{\mathbf{q}s,\mathbf{q}s} = -\tau^{-1}_{\mathbf{q}s,\mathrm{el-ph}}$ and

$$\tau^{-1}_{\mathbf{q}s,\mathrm{el-ph}} = \frac{4\pi}{\hbar} \sum_{mn} \langle |g_{mn,s}(\mathbf{k},\mathbf{q})|^2 (f_{n\mathbf{k}} - f_{m\mathbf{k}+\mathbf{q}}) \delta(\varepsilon_{m\mathbf{k}+\mathbf{q}} - \varepsilon_{n\mathbf{k}} - \hbar\omega_{\mathbf{q}s}) \rangle_{\mathrm{BZ}} \tag{19}$$

where f is the electron distribution function. In this case, Fermi-Dirac distribution is used for the assumption that only phonons are perturbed.

To study the hot carrier dynamics, we apply Allen's theory to calculate mode-resolved electron energy decay rate. When some of high-energy electron modes are excited, their energy exchange with phonon will eventually determine the rate of reaching equilibrium between electrons and phonons. For electron mode $n\mathbf{k}$, the energy decay rate due to phonon-electron scattering should be the result of integrating all possible combination $\mathbf{q}s + n\mathbf{k} \leftrightarrow m(\mathbf{k}+\mathbf{q})$ and $n\mathbf{k} \leftrightarrow m(\mathbf{k}+\mathbf{q}) + (-\mathbf{q})s$, leading to,

$$\left(\frac{\partial E_{n\mathbf{k}}}{\partial t}\right)_{\mathrm{ep}} = \frac{4\pi}{\hbar}\sum_{ms} \langle \hbar\omega_{\mathbf{q}s} \left| g_{mn,s}(\mathbf{k},\mathbf{q}) \right|^2 \left[-\left(n_{\mathbf{q}s} + f_{m\mathbf{k}+\mathbf{q}}\right)\delta\left(\varepsilon_{m\mathbf{k}+\mathbf{q}} - \varepsilon_{n\mathbf{k}} - \hbar\omega_{\mathbf{q}s}\right) + (n_{\mathbf{q}s} + 1 - f_{m\mathbf{k}+\mathbf{q}})\delta\left(\varepsilon_{m\mathbf{k}+\mathbf{q}} - \varepsilon_{n\mathbf{k}} + \hbar\omega_{\mathbf{q}s}\right)\right]\rangle_{\mathrm{BZ}} \tag{20}$$

The electron-phonon calculations were performed by using fully relativistic norm-conserving Vanderbilt pseudopotentials from the PseudoDojo project (*95*, *96*), with spin-orbit coupling included to capture fine electronic structures near the Fermi level. An energy cutoff of 100 Ry was used to ensure convergence. We first performed DFT and density functional perturbation theory (DFPT) calculations on 6 × 6 × 6 coarse **q**-mesh and 12 × 12 × 12 **k**-mesh, which were then interpolated to dense 72 × 72 × 72 **q**- and **k**-meshes using maximally localized Wannier interpolation technique through the embedded interface between EPW and Wannier90 (*97*). We used 20 Wannier bands, with the initial projections generated by the selected columns of the density matrix (SCDM). The interpolated electron and phonon band structures were carefully examined to be consistent with direct calculation using DFT. The electron-phonon coupling strength, Eliashberg function, electron-phonon scattering rate, and electron energy decay rate were calculated using our in-house software based on Wannier-interpolated data generated by EPW (*98*). The tetrahedron scheme (*94*) is applied for both **q**- and **k**-meshes.

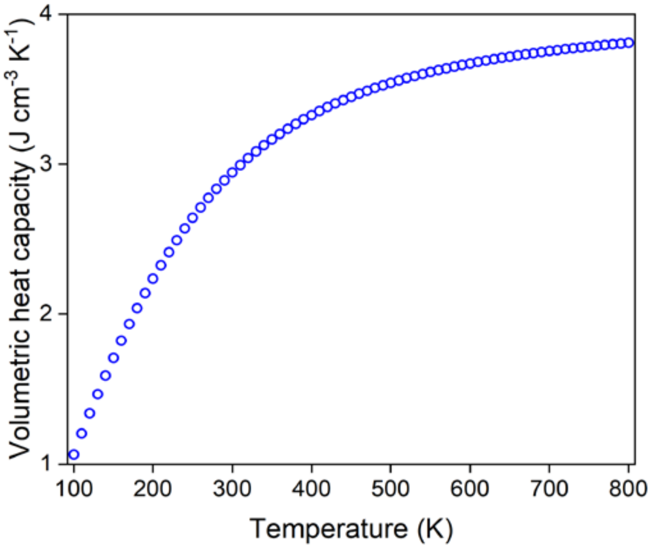

Fig. S1. Temperature-dependent volumetric heat capacity of θ-TaN.

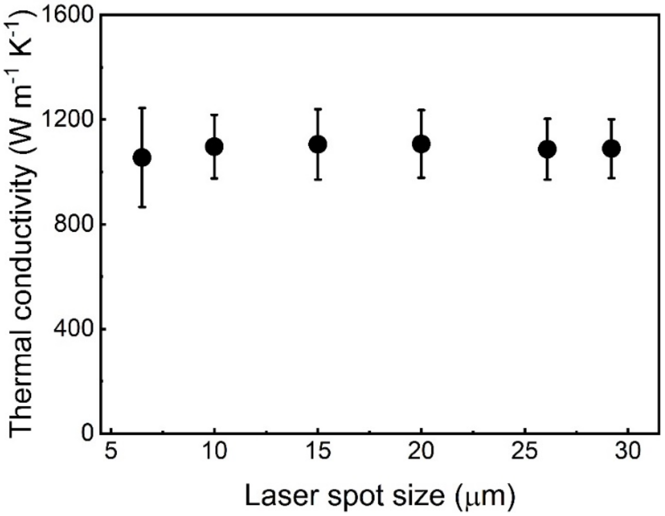

Fig. S2. Thermal measurements with varied laser spot sizes.

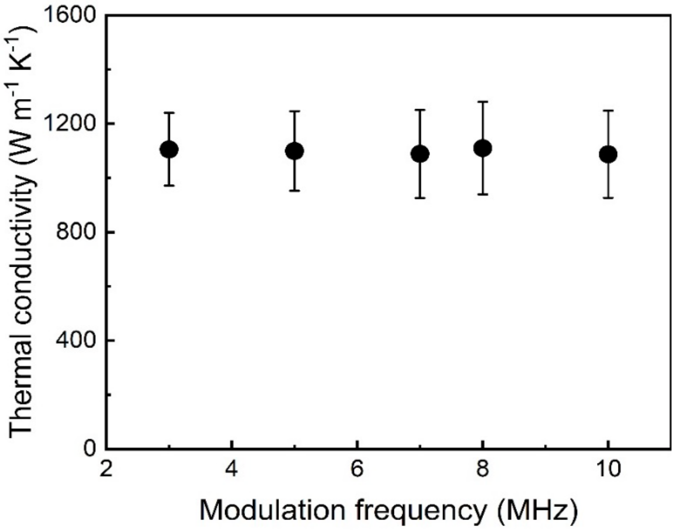

Fig. S3. Thermal measurements with varied modulation frequencies.

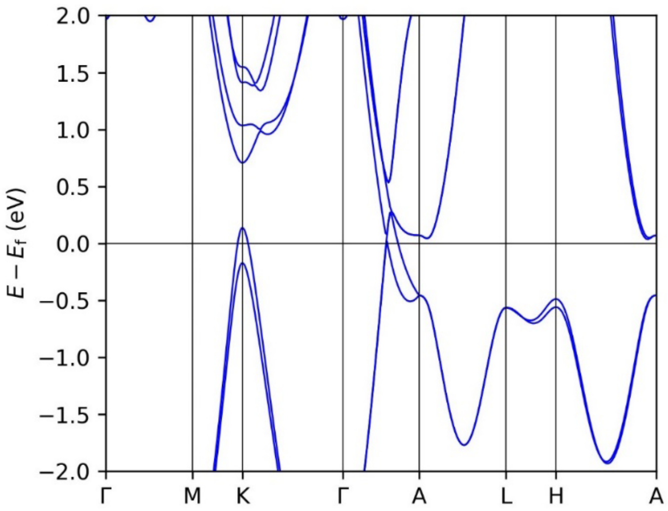

Fig. S4. Electronic band structure of θ-TaN.

References and Notes

1. M. Li, S. Li, Z. Zhang, C. Su, B. Wong, Y. Hu, Advancing thermal management technology for power semiconductors through materials and interface engineering. *Acc. Mater. Res.* **6**, 563–576 (2025). doi:10.1021/accountsmr.4c00349 Medline

2. S. Chu, A. Majumdar, Opportunities and challenges for a sustainable energy future. *Nature* **488**, 294–303 (2012). doi:10.1038/nature11475 Medline

3. A. Shehabi, A. Newkirk, S. J. Smith, A. Hubbard, N. Lei, M. A. B. Siddik, B. Holecek, J. Koomey, E. Masanet, D. Sartor, "2024 United States Data Center Energy Usage Report," Report No. LBNL-2001637 (Lawrence Berkeley National Laboratory, 2024); https://doi.org/10.71468/P1WC7Q.

4. "International Technology Roadmap for Semiconductors 2024 Edition" (2024); https://irds.ieee.org/editions/2024.

5. Y. Cui, M. Li, Y. Hu, Emerging interface materials for electronics thermal management: Experiments, modeling, and new opportunities. *J. Mater. Chem. C* **8**, 10568–10586 (2020). doi:10.1039/C9TC05415D

6. Global Insight Services, "Heat Sinks Market – Global Industry Analysis, Size, Share, Growth, Trends, and Forecast 2023–2032" (Global Insight Services, 2023).

7. J. M. Ziman, *Electrons and Phonons: The Theory of Transport Phenomena in Solids* (Oxford Univ. Press, 1960).

8. J. Bardeen, D. Pines, Electron-phonon interaction in metals. *Phys. Rev.* **99**, 1140–1150 (1955). doi:10.1103/PhysRev.99.1140

9. R. E. Prange, L. P. Kadanoff, Transport theory for electron-phonon interactions in metals. *Phys. Rev.* **134**, A566–A580 (1964). doi:10.1103/PhysRev.134.A566

10. L. Lindsay, D. A. Broido, T. L. Reinecke, First-principles determination of ultrahigh thermal conductivity of boron arsenide: A competitor for diamond? *Phys. Rev. Lett.* **111**, 025901 (2013). doi:10.1103/PhysRevLett.111.025901 Medline

11. T. Feng, L. Lindsay, X. Ruan, Four-phonon scattering significantly reduces intrinsic thermal conductivity of solids. *Phys. Rev. B* **96**, 161201 (2017). doi:10.1103/PhysRevB.96.161201

12. C. Dames, Ultrahigh thermal conductivity confirmed in boron arsenide. *Science* **361**, 549–550 (2018). doi:10.1126/science.aau4793 Medline

13. S. Li, Z. Qin, H. Wu, M. Li, M. Kunz, A. Alatas, A. Kavner, Y. Hu, Anomalous thermal transport under high pressure in boron arsenide. *Nature* **612**, 459–464 (2022). doi:10.1038/s41586-022-05381-x Medline

14. J. S. Kang, M. Li, H. Wu, H. Nguyen, Y. Hu, Experimental observation of high thermal conductivity in boron arsenide. *Science* **361**, 575–578 (2018). doi:10.1126/science.aat5522 Medline

15. S. Li, Q. Zheng, Y. Lv, X. Liu, X. Wang, P. Y. Huang, D. G. Cahill, B. Lv, High thermal conductivity in cubic boron arsenide crystals. *Science* **361**, 579–581 (2018). doi:10.1126/science.aat8982 Medline

16. F. Tian, B. Song, X. Chen, N. K. Ravichandran, Y. Lv, K. Chen, S. Sullivan, J. Kim, Y. Zhou, T.-H. Liu, M. Goni, Z. Ding, J. Sun, G. A. G. Udalamatta Gamage, H. Sun, H. Ziyaee, S. Huyan, L. Deng, J. Zhou, A. J. Schmidt, S. Chen, C.-W. Chu, P. Y. Huang, D. Broido, L. Shi, G. Chen, Z. Ren, Unusual high thermal conductivity in boron arsenide bulk crystals. *Science* **361**, 582–585 (2018). doi:10.1126/science.aat7932 Medline

17. J. S. Kang, M. Li, H. Wu, H. Nguyen, T. Aoki, Y. Hu, Integration of boron arsenide cooling substrates into gallium nitride devices. *Nat. Electron.* **4**, 416–423 (2021). doi:10.1038/s41928-021-00595-9

18. Y. Cui, Z. Qin, H. Wu, M. Li, Y. Hu, Flexible thermal interface based on self-assembled boron arsenide for high-performance thermal management. *Nat. Commun.* **12**, 1284 (2021). doi:10.1038/s41467-021-21531-7 Medline

19. N. Mingo, D. A. Broido, Lattice thermal conductivity crossovers in semiconductor nanowires. *Phys. Rev. Lett.* **93**, 246106 (2004). doi:10.1103/PhysRevLett.93.246106 Medline

20. N. Mingo, D. A. Broido, Carbon nanotube ballistic thermal conductance and its limits. *Phys. Rev. Lett.* **95**, 096105 (2005). doi:10.1103/PhysRevLett.95.096105 Medline

21. T. Wang, J. Carrete, A. Van Roekeghem, N. Mingo, G. K. H. Madsen, *Ab initio* phonon scattering by dislocations. *Phys. Rev. B* **95**, 245304 (2017). doi:10.1103/PhysRevB.95.245304

22. H. Fan, H. Wu, L. Lindsay, Y. Hu, *Ab initio* investigation of single-layer high thermal conductivity boron compounds. *Phys. Rev. B* **100**, 085420 (2019). doi:10.1103/PhysRevB.100.085420

23. H. Wu, H. Fan, Y. Hu, *Ab initio* determination of ultrahigh thermal conductivity in ternary compounds. *Phys. Rev. B* **103**, L041203 (2021). doi:10.1103/PhysRevB.103.L041203

24. M. Li, H. Wu, E. M. Avery, Z. Qin, D. P. Goronzy, H. D. Nguyen, T. Liu, P. S. Weiss, Y. Hu, Electrically gated molecular thermal switch. *Science* **382**, 585–589 (2023). doi:10.1126/science.abo4297 Medline

25. A. Kundu, X. Yang, J. Ma, T. Feng, J. Carrete, X. Ruan, G. K. H. Madsen, W. Li, Ultrahigh thermal conductivity of θ-phase tantalum nitride. *Phys. Rev. Lett.* **126**, 115901 (2021). doi:10.1103/PhysRevLett.126.115901 Medline

26. A. Kundu, Y. Chen, X. Yang, F. Meng, J. Carrete, M. Kabir, G. K. H. Madsen, W. Li, Electron-induced nonmonotonic pressure dependence of the lattice thermal conductivity of θ-TaN. *Phys. Rev. Lett.* **132**, 116301 (2024). doi:10.1103/PhysRevLett.132.116301 Medline

27. C. Li, D. Broido, Large electron-phonon drag asymmetry and reverse heat flow in the topological semimetal θ-TaN. *Mater. Today Phys.* **53**, 101706 (2025). doi:10.1016/j.mtphys.2025.101706

28. G. Brauer, K. H. Zapp, Die Nitride des Tantals. *Z. Anorg. Allg. Chem.* **277**, 129–139 (1954). doi:10.1002/zaac.19542770304

29. N. Schönberg, W. G. Overend, A. Munthe-Kaas, N. A. Sörensen, An X-ray study of the tantalum-nitrogen system. *Acta Chem. Scand.* **8**, 199–203 (1954). doi:10.3891/acta.chem.scand.08-0199

30. G. Brauer, E. Mohr, A. Neuhaus, A. Skokan, ϑ-TaN, eine Hochdruckform von Tantalnitrid. *Monatsh. Chem.* **103**, 794–798 (1972). doi:10.1007/BF00905439

31. N. Terao, Structure of tantalum nitrides. *Jpn. J. Appl. Phys.* **10**, 248 (1971). doi:10.1143/JJAP.10.248

32. J. Gatterer, G. Dufek, P. Ettmayer, R. Kieffer, Das kubische Tantalmononitrid (B 1-Typ) und seine Mischbarkeit mit den isotypen Übergangsmetallnitriden und-carbiden. *Monatsh. Chem.* **106**, 1137–1147 (1975). doi:10.1007/BF00906226

33. K. Frisk, Analysis of the phase diagram and thermochemistry in the Ta–N and the Ta–C–N systems. *J. Alloys Compd.* **278**, 216–226 (1998). doi:10.1016/S0925-8388(98)00582-9

34. A. Friedrich, W. Morgenroth, L. Bayarjargal, E. A. Juarez-Arellano, B. Winkler, Z. Konôpková, *In situ* study of the high pressure high-temperature stability field of TaN and of the compressibilities of ϑ-TaN and TaON. *High Press. Res.* **33**, 633–641 (2013). doi:10.1080/08957959.2013.813943

35. Eh. K. Molodovskaya, V. F. Petrunin, I. Karimov, Neutron-diffraction study of cubic tantalum nitride. *Phys. Met. Metallogr.* **40**, 202–204 (1975).

36. T. Mashimo, S. Tashiro, M. Nishida, K. Miyahara, E. Eto, B1-type and WC-type phase bulk bodies of tantalum nitride prepared by shock and static compressions. *Physica B* **239**, 13–15 (1997). doi:10.1016/S0921-4526(97)00367-0

37. H. Yusa, F. Kawamura, T. Taniguchi, N. Hirao, Y. Ohishi, T. Kikegawa, High-pressure synthesis and compressive behavior of tantalum nitrides. *J. Appl. Phys.* **115**, 103520 (2014). doi:10.1063/1.4867986

38. T. Mashimo, S. Tashiro, T. Toya, M. Nishida, H. Yamazaki, S. Yamaya, K. Oh-Ishi, Y. Syono, Synthesis of the B1-type tantalum nitride by shock compression. *J. Mater. Sci.* **28**, 3439–3443 (1993). doi:10.1007/BF01159819

39. I. P. Parkin, A. T. Rowley, Solid-state routes to tantalum nitrides (TaN, Ta_3N_5). *Adv. Mater.* **6**, 780–782 (1994). doi:10.1002/adma.19940061015

40. H. Lee, Y. Zhou, S. Jung, H. Li, Z. Cheng, J. He, J. Chen, P. Sokalski, A. Dolocan, R. Gearba-Dolocan, K. C. Matthews, F. Giustino, J. Zhou, L. Shi, High-pressure synthesis and thermal conductivity of semimetallic θ-tantalum nitride. *Adv. Funct. Mater.* **33**, 2212957 (2023). doi:10.1002/adfm.202212957

41. Y. Liu, Q. Li, Y. Qian, Y. Yang, S. Wang, W. Li, B. Sun, Thermal conductivity of high-temperature high-pressure synthesized θ-TaN. *Appl. Phys. Lett.* **122**, 222201 (2023). doi:10.1063/5.0146492

42. B. E. Warren, *X-Ray Diffraction* (Addison-Wesley, 1969).

43. M. Li, J. S. Kang, H. D. Nguyen, H. Wu, T. Aoki, Y. Hu, Anisotropic thermal boundary resistance across 2D black phosphorus: Experiment and atomistic modeling of interfacial

energy transport. *Adv. Mater.* **31**, e1901021 (2019). doi:10.1002/adma.201901021 Medline

44. J. S. Kang, H. Wu, Y. Hu, Thermal properties and phonon spectral characterization of synthetic boron phosphide for high thermal conductivity applications. *Nano Lett.* **17**, 7507–7514 (2017). doi:10.1021/acs.nanolett.7b03437 Medline

45. Y. Hu, L. Zeng, A. J. Minnich, M. S. Dresselhaus, G. Chen, Spectral mapping of thermal conductivity through nanoscale ballistic transport. *Nat. Nanotechnol.* **10**, 701–706 (2015). doi:10.1038/nnano.2015.109 Medline

46. M. Li, J. S. Kang, Y. Hu, Anisotropic thermal conductivity measurement using a new asymmetric-beam time-domain thermoreflectance (AB-TDTR) method. *Rev. Sci. Instrum.* **89**, 084901 (2018). doi:10.1063/1.5026028 Medline

47. N. W. Ashcroft, N. D. Mermin, *Solid State Physics* (Saunders College, 1976).

48. P. B. Allen, R. C. Dynes, Transition temperature of strong-coupled superconductors reanalyzed. *Phys. Rev. B* **12**, 905–922 (1975). doi:10.1103/PhysRevB.12.905

49. P. B. Allen, Theory of thermal relaxation of electrons in metals. *Phys. Rev. Lett.* **59**, 1460–1463 (1987). doi:10.1103/PhysRevLett.59.1460 Medline

50. C. Y. Ho, R. W. Powell, P. E. Liley, Thermal conductivity of the elements. *J. Phys. Chem. Ref. Data* **1**, 279–421 (1972). doi:10.1063/1.3253100

51. E. Bauer, C. Paul, S. Berger, S. Majumdar, H. Michor, M. Giovannini, A. Saccone, A. Bianconi, Thermal conductivity of superconducting MgB_2. *J. Phys. Condens. Matter* **13**, L487–L493 (2001). doi:10.1088/0953-8984/13/22/107

52. A. Kundu, J. Ma, J. Carrete, G. K. H. Madsen, W. Li, Anomalously large lattice thermal conductivity in metallic tungsten carbide and its origin in the electronic structure. *Mater. Today Phys.* **13**, 100214 (2020). doi:10.1016/j.mtphys.2020.100214

53. T. Ouyang, H. Xiao, C. Tang, M. Hu, J. Zhong, Anisotropic thermal transport in Weyl semimetal TaAs: A first principles calculation. *Phys. Chem. Chem. Phys.* **18**, 16709–16714 (2016). doi:10.1039/C6CP02935C Medline

54. Y. Zhou, Y.-Q. Zhao, Z.-Y. Zeng, X.-R. Chen, H.-Y. Geng, Anisotropic thermoelectric properties of Weyl semimetal NbX (X = P and As): A potential thermoelectric material. *Phys. Chem. Chem. Phys.* **21**, 15167–15176 (2019). doi:10.1039/C9CP02020A Medline

55. S.-D. Guo, Anisotropic lattice thermal conductivity in three-fold degeneracy topological semimetal MoP: A first-principles study. *J. Phys. Condens. Matter* **29**, 435704 (2017). doi:10.1088/1361-648X/aa8939 Medline

56. M. K. Hooda, C. S. Yadav, Electronic transport properties of intermediately coupled superconductors: $PdTe_2$ and $Cu_{0.04}PdTe_2$. *Europhys. Lett.* **121**, 17001 (2018). doi:10.1209/0295-5075/121/17001

57. Y. Chen, J. Ma, S. Wen, W. Li, Body-centered-cubic structure and weak anharmonic phonon scattering in tungsten. *NPJ Comput. Mater.* **5**, 98 (2019). doi:10.1038/s41524-019-0235-7

58. J. M. Rowell, W. L. McMillan, W. L. Feldmann, Superconductivity and lattice dynamics of white tin. *Phys. Rev. B* **3**, 4065–4073 (1971). doi:10.1103/PhysRevB.3.4065

59. S. Y. Savrasov, D. Y. Savrasov, Electron-phonon interactions and related physical properties of metals from linear-response theory. *Phys. Rev. B* **54**, 16487–16501 (1996). doi:10.1103/PhysRevB.54.16487 Medline

60. Y. Kong, O. V. Dolgov, O. Jepsen, O. K. Andersen, Electron-phonon interaction in the normal and superconducting states of MgB_2. *Phys. Rev. B* **64**, 020501 (2001). doi:10.1103/PhysRevB.64.020501

61. J. Chen, J. Gao, Strong electron–phonon coupling in 3D WN and coexistence of intrinsic superconductivity and topological nodal line in its 2D limit. *Phys. Status Solidi Rapid Res. Lett.* **16**, 2100477 (2022). doi:10.1002/pssr.202100477

62. S. Han, Q. Tang, H. Yuan, Y. Luo, H. Liu, Effects of electron-phonon coupling on the phonon transport properties of the Weyl semimetals NbAs and TaAs: A comparative study. *J. Materiomics* **9**, 520–526 (2023). doi:10.1016/j.jmat.2022.12.001

63. K. T. Chan, B. D. Malone, M. L. Cohen, Electron-phonon coupling and superconductivity in arsenic under pressure. *Phys. Rev. B* **86**, 094515 (2012). doi:10.1103/PhysRevB.86.094515

64. J. Chen, Unconventional superconductivity in the topological semimetal MoP: Evidence from first-principles calculated electron–phonon coupling. *Comput. Mater. Sci.* **173**, 109466 (2020). doi:10.1016/j.commatsci.2019.109466

65. G. Anemone, P. Casado Aguilar, M. Garnica, F. Calleja, A. Al Taleb, C.-N. Kuo, C. S. Lue, A. Politano, A. L. Vázquez De Parga, G. Benedek, D. Farías, R. Miranda, Electron–phonon coupling in superconducting 1T-$PdTe_2$. *NPJ 2D Mater. Appl.* **5**, 25 (2021). doi:10.1038/s41699-021-00204-5

66. M. Alcántara Ortigoza, I. Y. Sklyadneva, R. Heid, E. V. Chulkov, T. S. Rahman, K.-P. Bohnen, P. M. Echenique, *Ab initio* lattice dynamics and electron-phonon coupling of Bi(111). *Phys. Rev. B* **90**, 195438 (2014). doi:10.1103/PhysRevB.90.195438

67. N. Kuge, T. Sekiya, M. Enoki, H. Yamane, T. Yamada, Preparation of faceted TaN grains by heating Ta-containing oxides in BN crucible with Na. *Ceram. Int.* **48**, 10817–10820 (2022). doi:10.1016/j.ceramint.2021.12.297

68. M. Kunz, A. A. MacDowell, W. A. Caldwell, D. Cambie, R. S. Celestre, E. E. Domning, R. M. Duarte, A. E. Gleason, J. M. Glossinger, N. Kelez, D. W. Plate, T. Yu, J. M. Zaug, H. A. Padmore, R. Jeanloz, A. P. Alivisatos, S. M. Clark, A beamline for high-pressure studies at the Advanced Light Source with a superconducting bending magnet as the source. *J. Synchrotron Radiat.* **12**, 650–658 (2005). doi:10.1107/S0909049505020959 Medline

69. A. H. Said, H. Sinn, T. S. Toellner, E. E. Alp, T. Gog, B. M. Leu, S. Bean, A. Alatas, High-energy-resolution inelastic X-ray scattering spectrometer at beamline 30-ID of the Advanced Photon Source. *J. Synchrotron Radiat.* **27**, 827–835 (2020). doi:10.1107/S1600577520002854 Medline

70. T. S. Toellner, A. Alatas, A. H. Said, Six-reflection meV-monochromator for synchrotron radiation. *J. Synchrotron Radiat.* **18**, 605–611 (2011). doi:10.1107/S0909049511017535 Medline

71. A. J. Schmidt, X. Chen, G. Chen, Pulse accumulation, radial heat conduction, and anisotropic thermal conductivity in pump-probe transient thermoreflectance. *Rev. Sci. Instrum.* **79**, 114902 (2008). doi:10.1063/1.3006335 Medline

72. D. G. Cahill, Analysis of heat flow in layered structures for time-domain thermoreflectance. *Rev. Sci. Instrum.* **75**, 5119–5122 (2004). doi:10.1063/1.1819431

73. G. T. Hohensee, R. B. Wilson, D. G. Cahill, Thermal conductance of metal-diamond interfaces at high pressure. *Nat. Commun.* **6**, 6578 (2015). doi:10.1038/ncomms7578 Medline

74. A. Sood, J. Cho, K. D. Hobart, T. I. Feygelson, B. B. Pate, M. Asheghi, D. G. Cahill, K. E. Goodson, Anisotropic and inhomogeneous thermal conduction in suspended thin-film polycrystalline diamond. *J. Appl. Phys.* **119**, 175103 (2016). doi:10.1063/1.4948335

75. J. S. Kang, M. Ke, Y. Hu, Ionic intercalation in two-dimensional van der Waals materials: In situ characterization and electrochemical control of the anisotropic thermal conductivity of black phosphorus. *Nano Lett.* **17**, 1431–1438 (2017). doi:10.1021/acs.nanolett.6b04385 Medline

76. Z. Qin, L. Dai, M. Li, S. Li, H. Wu, K. E. White, G. Gani, P. S. Weiss, Y. Hu, Moiré pattern controlled phonon polarizer based on twisted graphene. *Adv. Mater.* **36**, e2312176 (2024). doi:10.1002/adma.202312176 Medline

77. M. N. Luckyanova, J. Garg, K. Esfarjani, A. Jandl, M. T. Bulsara, A. J. Schmidt, A. J. Minnich, S. Chen, M. S. Dresselhaus, Z. Ren, E. A. Fitzgerald, G. Chen, Coherent phonon heat conduction in superlattices. *Science* **338**, 936–939 (2012). doi:10.1126/science.1225549 Medline

78. J. P. Feser, D. G. Cahill, Probing anisotropic heat transport using time-domain thermoreflectance with offset laser spots. *Rev. Sci. Instrum.* **83**, 104901 (2012). doi:10.1063/1.4757863 Medline

79. K. Chen, B. Song, N. K. Ravichandran, Q. Zheng, X. Chen, H. Lee, H. Sun, S. Li, G. A. G. Udalamatta Gamage, F. Tian, Z. Ding, Q. Song, A. Rai, H. Wu, P. Koirala, A. J. Schmidt, K. Watanabe, B. Lv, Z. Ren, L. Shi, D. G. Cahill, T. Taniguchi, D. Broido, G. Chen, Ultrahigh thermal conductivity in isotope-enriched cubic boron nitride. *Science* **367**, 555–559 (2020). doi:10.1126/science.aaz6149 Medline

80. R. B. Wilson, J. P. Feser, G. T. Hohensee, D. G. Cahill, Two-channel model for nonequilibrium thermal transport in pump-probe experiments. *Phys. Rev. B* **88**, 144305 (2013). doi:10.1103/PhysRevB.88.144305

81. C. A. Paddock, G. L. Eesley, Transient thermoreflectance from thin metal films. *J. Appl. Phys.* **60**, 285–290 (1986). doi:10.1063/1.337642

82. X. Zheng, D. Cahill, P. Krasnochtchekov, R. Averback, J. Zhao, High-throughput thermal conductivity measurements of nickel solid solutions and the applicability of the

Wiedemann–Franz law. *Acta Mater.* **55**, 5177–5185 (2007). doi:10.1016/j.actamat.2007.05.037

83. J. S. Kang, H. Wu, M. Li, Y. Hu, Intrinsic low thermal conductivity and phonon renormalization due to strong anharmonicity of single-crystal tin selenide. *Nano Lett.* **19**, 4941–4948 (2019). doi:10.1021/acs.nanolett.9b01056 Medline

84. J. L. Braun, C. J. Szwejkowski, A. Giri, P. E. Hopkins, On the steady-state temperature rise during laser heating of multilayer thin films in optical pump–probe techniques. *J. Heat Transfer* **140**, 052801 (2018). doi:10.1115/1.4038713

85. P. Jiang, X. Qian, R. Yang, Tutorial: Time-domain thermoreflectance (TDTR) for thermal property characterization of bulk and thin film materials. *J. Appl. Phys.* **124**, 161103 (2018). doi:10.1063/1.5046944

86. H. E. Elsayed-Ali, T. B. Norris, M. A. Pessot, G. A. Mourou, Time-resolved observation of electron-phonon relaxation in copper. *Phys. Rev. Lett.* **58**, 1212–1215 (1987). doi:10.1103/PhysRevLett.58.1212 Medline

87. R. W. Schoenlein, W. Z. Lin, J. G. Fujimoto, G. L. Eesley, Femtosecond studies of nonequilibrium electronic processes in metals. *Phys. Rev. Lett.* **58**, 1680–1683 (1987). doi:10.1103/PhysRevLett.58.1680 Medline

88. S. D. Brorson, A. Kazeroonian, J. S. Moodera, D. W. Face, T. K. Cheng, E. P. Ippen, M. S. Dresselhaus, G. Dresselhaus, Femtosecond room-temperature measurement of the electron-phonon coupling constant γ in metallic superconductors. *Phys. Rev. Lett.* **64**, 2172–2175 (1990). doi:10.1103/PhysRevLett.64.2172 Medline

89. H. Wu, Z. Qin, S. Li, L. Lindsay, Y. Hu, Nonperturbative determination of isotope-induced anomalous vibrational physics. *Phys. Rev. B* **108**, L140302 (2023). doi:10.1103/PhysRevB.108.L140302 Medline

90. P. Giannozzi, S. Baroni, N. Bonini, M. Calandra, R. Car, C. Cavazzoni, D. Ceresoli, G. L. Chiarotti, M. Cococcioni, I. Dabo, A. Dal Corso, S. de Gironcoli, S. Fabris, G. Fratesi, R. Gebauer, U. Gerstmann, C. Gougoussis, A. Kokalj, M. Lazzeri, L. Martin-Samos, N. Marzari, F. Mauri, R. Mazzarello, S. Paolini, A. Pasquarello, L. Paulatto, C. Sbraccia, S. Scandolo, G. Sclauzero, A. P. Seitsonen, A. Smogunov, P. Umari, R. M. Wentzcovitch, QUANTUM ESPRESSO: A modular and open-source software project for quantum simulations of materials. *J. Phys. Condens. Matter* **21**, 395502 (2009). doi:10.1088/0953-8984/21/39/395502 Medline

91. P. E. Blöchl, Projector augmented-wave method. *Phys. Rev. B* **50**, 17953–17979 (1994). doi:10.1103/PhysRevB.50.17953 Medline

92. J. P. Perdew, A. Zunger, Self-interaction correction to density-functional approximations for many-electron systems. *Phys. Rev. B* **23**, 5048–5079 (1981). doi:10.1103/PhysRevB.23.5048

93. T. Tadano, Y. Gohda, S. Tsuneyuki, Anharmonic force constants extracted from first-principles molecular dynamics: Applications to heat transfer simulations. *J. Phys. Condens. Matter* **26**, 225402 (2014). doi:10.1088/0953-8984/26/22/225402 Medline

94. P. E. Blöchl, O. Jepsen, O. K. Andersen, Improved tetrahedron method for Brillouin-zone integrations. *Phys. Rev. B* **49**, 16223–16233 (1994). doi:10.1103/PhysRevB.49.16223 Medline

95. D. R. Hamann, Optimized norm-conserving Vanderbilt pseudopotentials. *Phys. Rev. B* **88**, 085117 (2013). doi:10.1103/PhysRevB.88.085117

96. M. J. van Setten, M. Giantomassi, E. Bousquet, M. J. Verstraete, D. R. Hamann, X. Gonze, G.-M. Rignanese, The PseudoDojo: Training and grading a 85 element optimized norm-conserving pseudopotential table. *Comput. Phys. Commun.* **226**, 39–54 (2018). doi:10.1016/j.cpc.2018.01.012

97. G. Pizzi, V. Vitale, R. Arita, S. Blügel, F. Freimuth, G. Géranton, M. Gibertini, D. Gresch, C. Johnson, T. Koretsune, J. Ibañez-Azpiroz, H. Lee, J.-M. Lihm, D. Marchand, A. Marrazzo, Y. Mokrousov, J. I. Mustafa, Y. Nohara, Y. Nomura, L. Paulatto, S. Poncé, T. Ponweiser, J. Qiao, F. Thöle, S. S. Tsirkin, M. Wierzbowska, N. Marzari, D. Vanderbilt, I. Souza, A. A. Mostofi, J. R. Yates, Wannier90 as a community code: New features and applications. *J. Phys. Condens. Matter* **32**, 165902 (2020). doi:10.1088/1361-648X/ab51ff Medline

98. H. Lee, S. Poncé, K. Bushick, S. Hajinazar, J. Lafuente-Bartolome, J. Leveillee, C. Lian, J.-M. Lihm, F. Macheda, H. Mori, H. Paudyal, W. H. Sio, S. Tiwari, M. Zacharias, X. Zhang, N. Bonini, E. Kioupakis, E. R. Margine, F. Giustino, Electron–phonon physics from first principles using the EPW code. *NPJ Comput. Mater.* **9**, 156 (2023). doi:10.1038/s41524-023-01107-3
